# C/EBPβ deletion in macrophages impairs mammary gland alveolar budding during the estrous cycle

**DOI:** 10.26508/lsa.202302516

**Published:** 2024-07-18

**Authors:** Michelle D Rojo, Ishitri Bandyopadhyay, Caitlin M Burke, Alexa D Sturtz, Emily S Phillips, Megan G Matherne, Samuel J Embrey, Rebecca LaRue, Yinjie Qiu, Kathryn L Schwertfeger, Heather L Machado

**Affiliations:** 1 Department of Biochemistry and Molecular Biology, Tulane University School of Medicine, New Orleans, LA, USA; 2 Department of Laboratory Medicine and Pathology, Masonic Cancer Center, and Center for Immunology, University of Minnesota, Minneapolis, MN, USA; 3 Minnesota Supercomputing Institute, University of Minnesota, Minneapolis, MN, USA; 4 Tulane Cancer Center, Louisiana Cancer Research Consortium, New Orleans, LA, USA

## Abstract

Macrophages have important roles in mammary gland development and tissue homeostasis, but the specific mechanisms that regulate macrophage function need further elucidation. We have identified C/EBPβ as an important transcription factor expressed by multiple macrophage populations in the normal mammary gland. Mammary glands from mice with C/EBPβ-deficient macrophages (*Cebpb*^ΔM^) show a significant decrease in alveolar budding during the diestrus stage of the reproductive cycle, whereas branching morphogenesis remains unchanged. Defects in alveolar budding were found to be the result of both systemic hormones and local macrophage-directed signals. RNA sequencing shows significant changes in PR-responsive genes and alterations in the Wnt landscape of mammary epithelial cells of *Cebpb*^ΔM^ mice, which regulate stem cell expansion during diestrus. *Cebpb*^ΔM^ macrophages demonstrate a shift from a pro-inflammatory to a tissue-reparative phenotype, and exhibit increased phagocytic capacity as compared to WT. Finally, *Cebpb*^ΔM^ macrophages down-regulate *Notch2* and *Notch3*, which normally promote stem cell expansion during alveolar budding. These results suggest that C/EBPβ is an important macrophage factor that facilitates macrophage–epithelial crosstalk during a key stage of mammary gland tissue homeostasis.

## Abstract (precis)

Macrophage C/EBPβ contributes to alveolar budding during the estrous cycle by providing critical signaling to mammary epithelial cells.

## Introduction

Macrophages are specialized phagocytes that have important roles in development, tissue repair, and immune surveillance (1). These heterogeneous immune cells constitute an important component of the mammary gland by promoting development and maintaining tissue homeostasis. Macrophages are influenced by tissue-specific environmental cues, have unique cell programming, and regulate the formation and maintenance of epithelial structures during mammary gland development (2, 3, 4, 5, 6, 7). However, the specific mechanisms that regulate macrophage function, and how macrophage-derived factors contribute to mammary gland development have not been fully elucidated.

Mammary gland development is a dynamic process that occurs postnatally, and is tightly regulated by systemic hormones, local growth factors, and other circulating factors such as cytokines (6, 8, 9, 10, 11, 12). Numerous studies have indicated the contribution of stromal cells, including macrophages, to mammary gland remodeling during distinct developmental stages such as puberty, pregnancy, and involution. Throughout these stages, macrophages are closely associated with the ductal epithelium and thought to function in maintaining tissue homeostasis by clearing apoptotic debris and by providing key paracrine signals to the adjacent epithelium (13, 14, 15, 16, 17). Early studies showed that genetic ablation of macrophages results in impaired ductal elongation, terminal end bud (TEB) formation, and branching morphogenesis during puberty (4, 7), demonstrating the importance of macrophages to proper mammary gland function. Other studies revealed important functions for macrophages during the estrous cycle and involution (6, 18, 19), two periods of robust tissue remodeling where epithelial and stromal compartments reciprocally communicate key signals to restore and maintain homeostasis.

The transcription factor C/EBPβ (CCAAT/enhancer-binding protein beta) is a master regulator of proliferation and differentiation in various cell and tissue types, contributing to diverse biological and pathological processes including hematopoiesis, metabolism, inflammation, and tumorigenesis (20). In the mammary gland, C/EBPβ is highly expressed in the ductal epithelium and in other stromal cells such as adipocytes, fibroblasts, and immune cells. We and others have demonstrated epithelial-intrinsic functions of C/EBPβ during ductal elongation and pregnancy, as well as regulation of mammary stem and progenitor cell activity (21, 22, 23). Although it is well established that epithelial-derived C/EBPβ is a key regulator of mammary gland development, its function in mammary gland macrophages has not been studied. In other tissues, C/EBPβ is an important macrophage factor that is required for the differentiation of monocytes to macrophages and is crucial for proper phagocytic function (24, 25). Macrophage C/EBPβ is induced by pro-inflammatory cytokines, such as IL-6, TNFα, IFNγ, and IL-1β at sites of inflammation, and can in turn activate transcription of these genes, suggesting a pivotal role in modulating inflammation (26, 27). In contrast, C/EBPβ can induce arginase I expression, suggesting an anti-inflammatory, immune suppressive function in response to injury (28, 29). Although macrophage C/EBPβ is known to modulate the immune response and orchestrate tissue repair in a number of tissues, its contribution to mammary gland morphogenesis and homeostasis remains unknown.

Extensive studies have focused on understanding how tissue-specific extrinsic factors shape macrophage identity and function in a number of tissues; however, tissue-specific regulation of mammary gland macrophages has not been intensively investigated. Thus, we sought to identify key signaling pathways that regulate macrophage function during mammary gland development. We identified C/EBPβ as an important macrophage factor that provides crucial paracrine signals to adjacent epithelial cells to help maintain tissue homeostasis during the estrous cycle.

## Results

### C/EBPβ is expressed in mammary gland macrophages

**Figure 1.**
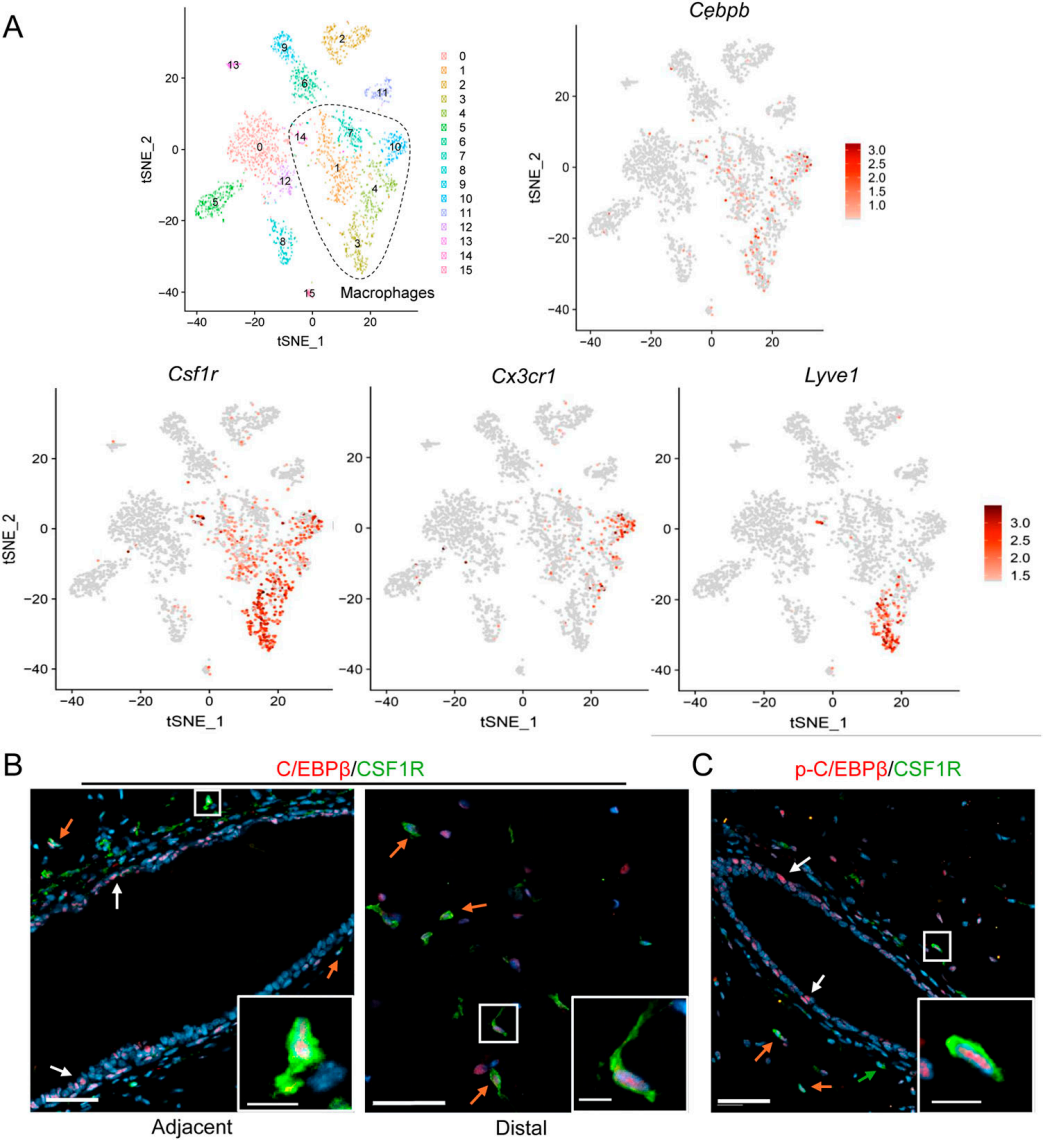
C/EBPβ is expressed in mammary gland macrophages. **(A)** t-SNE plot shows scRNA-seq analysis of CD45^+^ cells isolated from mammary glands of 10-wk-old FVB mice (30), where dotted line indicates putative macrophage populations. Feature plots depict *Cebpb*, *Csf1r*, *Cx3cr1*, and *Lyve1*. **(B)** Representative 20X images depict immunostaining of mammary glands from 10-wk-old diestrus-staged WT mice. CSF1R^+^ (green) and C/EBPβ^+^ (red) cells in the adjacent (left) and distal (right) stroma (n = 3). Orange arrows show macrophage C/EBPβ, and white arrows show epithelial C/EBPβ. Scale bar = 50 μm. Inset scale bar = 10 μm. **(C)** Representative 20X images of immunostaining for CSF1R (green) and p-C/EBPβ-Thr235 (red) in WT mammary glands (n = 3). Orange arrows show macrophage p-C/EBPβ, and white arrows show epithelial p-C/EBPβ. Scale bar = 50 μm. Inset scale bar = 10 μm.

Macrophages contribute to many stages of mammary gland development by promoting ductal elongation, ductal branching, ECM remodeling, and phagocytic clearance of apoptotic debris. To identify potential macrophage factors that modulate these processes, we recently performed single-cell RNA sequencing on CD45^+^ immune cells isolated from adult cycling female mice. Our studies and others revealed distinct ductal- and stromal-associated tissue-resident macrophage populations that contribute to maintaining the developing ductal structures in the mammary gland (18, 30, 31). MHCII^HI^CX3CR1^+^ macrophages are closely associated with the ductal epithelium (18), whereas LYVE-1^+^ macrophages localize to regions of hyaluronan throughout the stroma (30). Further analysis of the transcriptional profiles of these populations showed that *Cebpb* is highly expressed in *Csf1r*^*+*^ cells, including *Csf1r*^*+*^*Cx3cr1*^*+*^ (ductal) and *Csf1r*^*+*^*Lyve1*^*+*^ (stromal) macrophages (Fig 1A). In support of these findings, we previously reported that *Cebpb* is highly expressed in five distinct macrophage subsets in a syngeneic mouse model of early-stage breast cancer progression (32). To validate these data, paraffin-embedded mammary glands from adult WT mice were co-stained with antibodies to C/EBPβ and CSF1R. C/EBPβ^+^CSF1R^+^ cells were found in both the adjacent and distal stromal cells, suggesting that C/EBPβ may be important in various macrophage populations (Fig 1B). Furthermore, phosphorylated C/EBPβ (p-C/EBPβ), an indicator of transcriptional activity (33), was detected in CSF1R^+^ cells in both the adjacent (Fig 1C) and distal (data not shown) stromal cells. These results demonstrate that C/EBPβ is expressed and phosphorylated in CSF1R^+^ cells in the mammary gland.

**Figure 2.**
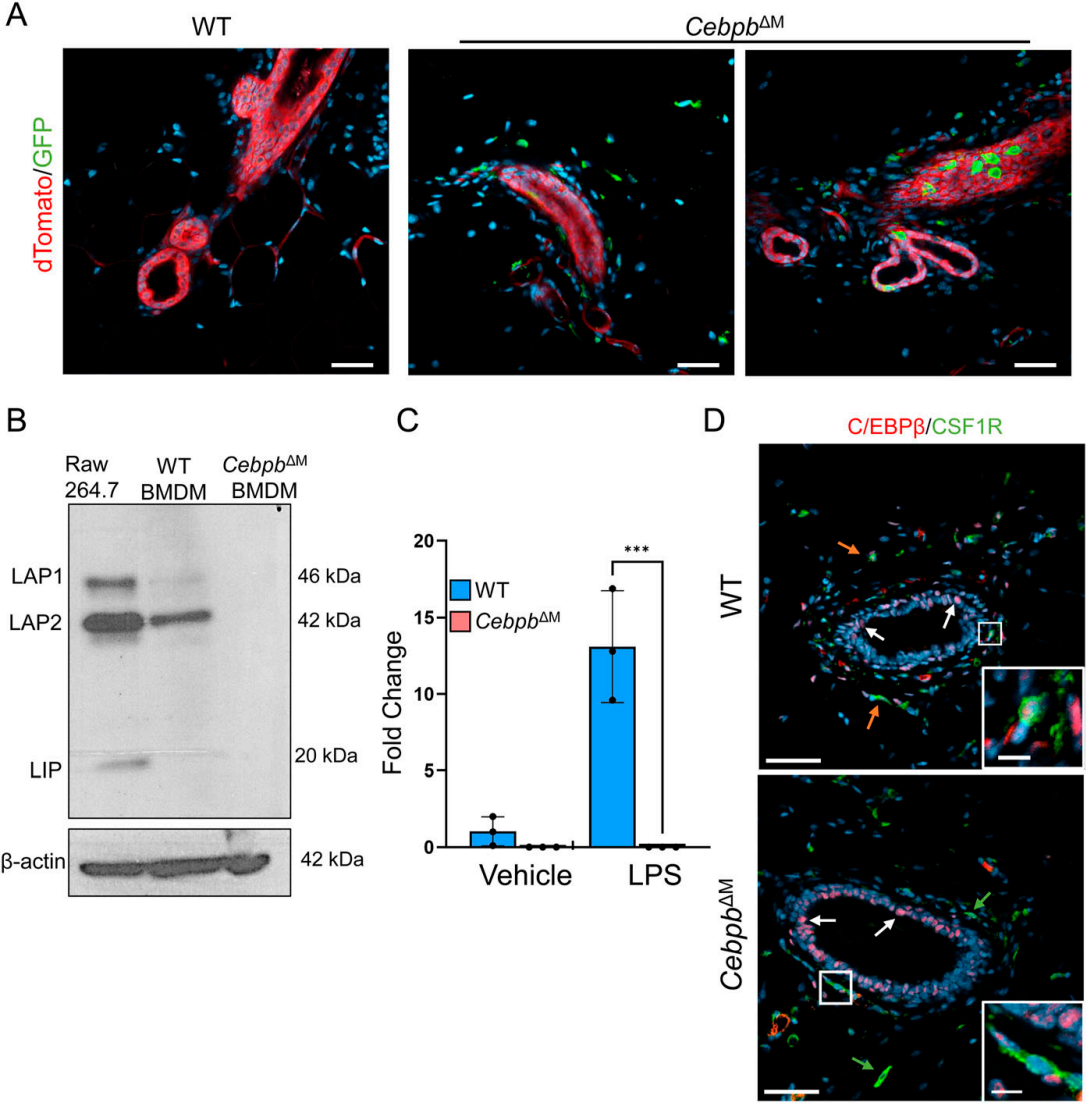
Deletion of *Cebpb* in macrophages. **(A)** Representative 40X confocal imaging of whole glands from WT and *Cebpb*^ΔM^ mice bred to Rosa^mTmG^ reporter mice. Cre^neg^ cells express dTomato (red), and cells that have undergone Cre recombination express GFP (green) (n = 3). Scale bar = 40 μm. **(B)** Immunoblot of C/EBPβ (LAP1, LAP2, LIP) in RAW 264.7 cells, WT BMDMs, and *Cebpb*^ΔM^ BMDMs (n = 3). **(C)** Graph shows qRT-PCR analysis of *Cebpb* from vehicle- or LPS-treated BMDMs from WT and *Cebpb*^ΔM^ mice (three mice per group). Values represent the mean and SD (***P = 0.0001, one-way ANOVA with multiple comparisons). **(D)** Representative 20X images depicting immunostaining for C/EBPβ (red) and CSF1R (green) in WT and *Cebpb*^ΔM^ mice (three mice per group). Orange arrows show macrophage C/EBPβ, white arrows show epithelial C/EBPβ, and green arrows show macrophages where C/EBPβ has been deleted. Scale bar = 50 μm. Inset scale bar = 10 μm.

In order to dissect the function of C/EBPβ in macrophages during mammary gland development, a conditional knockout model was generated, where C/EBPβ is deleted in CSF1R-expressing myeloid cells, hereafter referred to as macrophages. *Csf1r-iCre* mice were bred to *Cebpb*-floxed mice to conditionally delete *Cebpb* in CSF1R^+^ macrophages (*Cebpb*^ΔM^). To validate the efficiency of Cre recombination in vivo, *Cebpb*^ΔM^ mice were bred to mTmG reporter mice, where Cre^neg^ cells express dTomato red, and cells that have undergone Cre recombination express GFP. Whole-gland confocal imaging confirms the presence of dTomato red cells in the ductal epithelium and surrounding stromal cells in WT mice (*Csf1r-Cre*^*neg*^*;Cebpb*^*fl/fl*^), indicating the absence of Cre recombination. In contrast, *Cebpb*^ΔM^ mice exhibit dTomato red cells in the ductal epithelium and some stromal cells, whereas GFP^+^ cells are restricted to the adjacent or distal stroma, confirming the specificity of Cre recombination in the stromal cells that are likely macrophages (Fig 2A). GFP^+^ cells were also detected in close contact with ductal cells dispersed throughout the ducts in a fashion that was previously reported to be consistent with macrophages (18). Deletion of C/EBPβ was verified in BMDMs isolated from WT and *Cebpb*^ΔM^ mice and analyzed by immunoblotting, where C/EBPβ protein isoforms LAP1, LAP2, and LIP were not detectable in *Cebpb*^ΔM^ BMDMs (Fig 2B). Similarly, qRT-PCR analysis shows undetectable levels of *Cebpb* mRNA in *Cebpb*^ΔM^ BMDMs treated with LPS to induce *Cebpb* expression (Fig 2C). Finally, C/EBPβ deletion in the mammary gland was confirmed by co-immunostaining, where C/EBPβ is absent in CSF1R^+^ cells of *Cebpb*^ΔM^ mice but is expressed in epithelial and other stromal cells (Fig 2D). These results demonstrate that Cre expression and *Cebpb* deletion occur in macrophages.

### *Cebpb* deletion in macrophages impairs alveolar budding in the adult mammary gland

**Figure 3.**
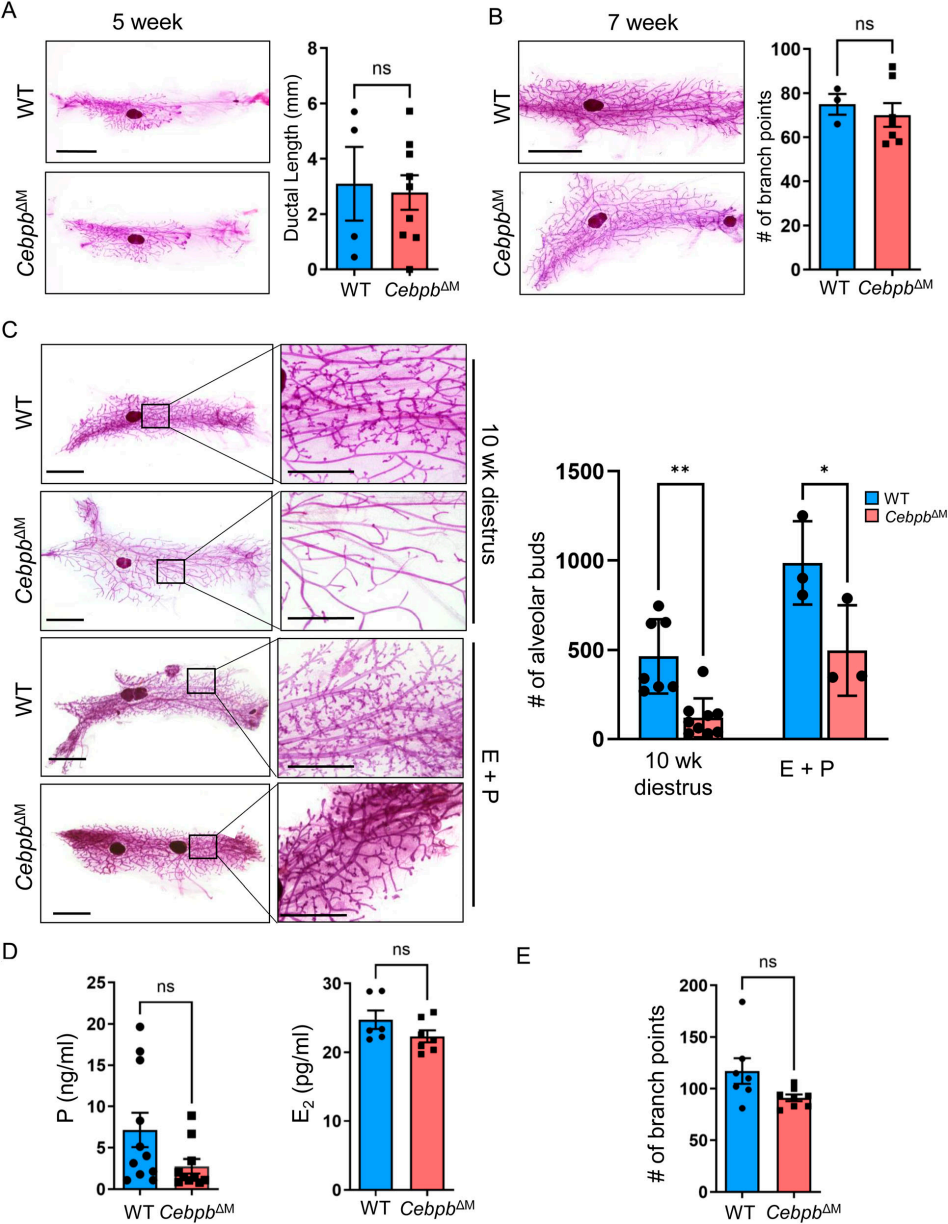
Cebpb deletion in macrophages impairs alveolar budding in the adult mammary gland. **(A)** Representative whole-mount images of carmine alum–stained mammary glands at 5 wk of age (scale bar = 5 mm) from WT and *Cebpb*^ΔM^ mice. The graph shows the quantification of ductal elongation by measuring the distance (mm) from the center of the lymph node to the most distal tip of a TEB (*P* = 0.80, unpaired *t* test) (four to eight mice per group). **(B)** Whole mounts of mammary glands from 7-wk-old animals harvested in diestrus (scale bar = 5 mm). The graph depicts the quantification of branching morphogenesis, analyzed by counting the branch points in the entire gland (*P* = 0.60, unpaired *t* test) (three to seven mice per group). **(C)** Left: whole mounts of mammary glands from 10-wk-old animals harvested in diestrus or after E+P treatment, showing changes in alveolar budding in *Cebpb*^ΔM^ glands as compared to WT (scale bar = 5 mm). Higher magnification (right), scale bar = 2 mm. Right: the graph depicts the quantification of alveolar budding after counting all alveolar buds in an entire gland (**P = 0.006, *P = 0.02, one-way ANOVA with multiple comparisons) (three to nine mice per group). **(D)** Plasma was collected from 10-wk-old WT and *Cebpb*^ΔM^ mice in diestrus and analyzed for progesterone (left) and estradiol (right). Graphs depict levels of progesterone in ng/ml (*P* = 0.08, unpaired *t* test) and estradiol in pg/ml (*P* = 0.15, unpaired *t* test) from 6 to 11 mice per group. **(E)** Quantification of branching morphogenesis (*P* = 0.51, unpaired *t* test) in 10-wk-old diestrus-staged animals from seven to nine mice per group.

**Figure S1.**
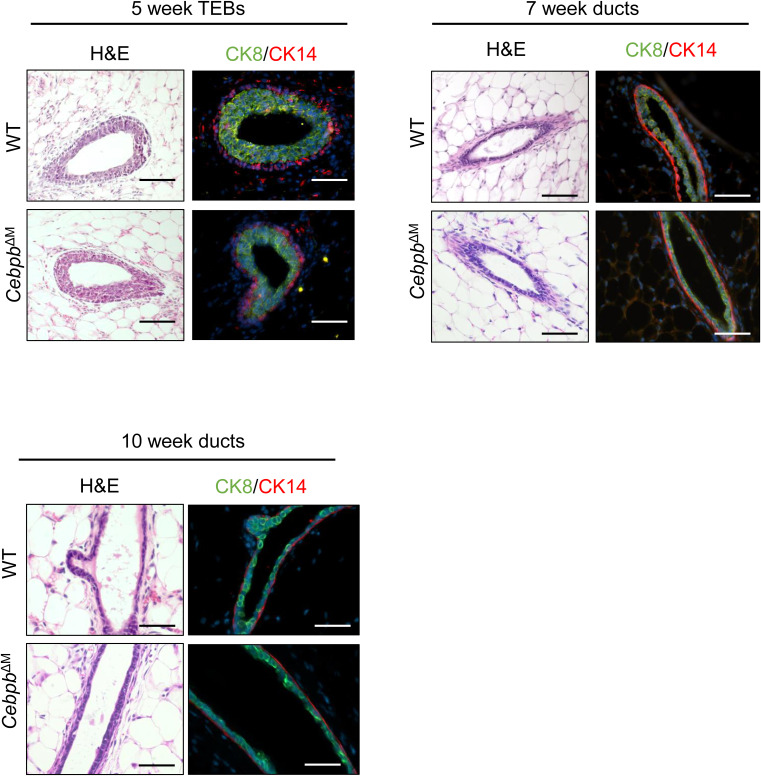
Cebpb^ΔM^ mice have normal ductal morphology. Representative 20X images depicting H&E staining and immunostaining (CK8: green; CK14: red) of TEBs of 5-wk-old mammary glands (top left), ducts of 7-wk-old mammary glands from diestrus-staged mice (top right), and ducts of 10-wk-old mammary glands from diestrus-staged mice (bottom left) (n = 3–6; scale bar = 40 μm).

Previous studies showed that genetic ablation of macrophages results in impaired ductal morphogenesis during puberty (4, 7). To assess the function of C/EBPβ in mammary gland macrophages during ductal morphogenesis, early stages of virgin mammary gland development were analyzed in WT and *Cebpb*^ΔM^ mice. Initial studies show that there are no changes in ductal elongation (5-wk-old mice) or branching morphogenesis (7-wk-old diestrus-staged mice) in *Cebpb*^ΔM^ as compared to WT mice (Fig 3A and B). Histological analysis and co-staining for luminal (cytokeratin [CK] 8) and myoepithelial (CK14) markers during these stages show intact luminal and basal epithelial layers in *Cebpb*^ΔM^ glands, with no visible alterations in the TEBs, ducts, or stromal cells (Fig S1). These results suggest that C/EBPβ is not required for macrophage-directed ductal morphogenesis during puberty.

Macrophages also have important functions in maintaining tissue homeostasis throughout the estrous cycle, where robust tissue modeling occurs particularly in the diestrus phase. During the diestrus stage of the reproductive cycle, macrophages promote alveolar bud formation to prepare for milk production if a pregnancy occurs. In the absence of pregnancy, macrophages help facilitate alveolar bud regression as the mice enter the pro-estrous stage of the cycle (2, 34). Analysis of mammary glands from diestrus-staged adult mice shows that alveolar budding is significantly decreased in *Cebpb*^ΔM^ mice as compared to WT littermate controls, strikingly resembling estrous-staged mammary glands (Fig 3C). In addition to local macrophage-derived signals, alveolar bud formation during diestrus is regulated by systemic hormone signaling (34, 35). To determine whether the observed budding defects are due to changes in systemic hormones, mice were treated with exogenous estradiol and progesterone (E+P). Administration of E+P partially restores alveolar budding in *Cebpb*^ΔM^ mammary glands (Fig 3C), suggesting that both local and systemic cues regulate alveolar budding. Because circulating progesterone levels peak during diestrus and promote alveolar budding and ductal side-branching (35, 36, 37), circulating estradiol and progesterone levels were analyzed. Although there are no significant differences in serum hormone levels between WT and *Cebpb*^ΔM^ mice, there is a trend for reduced progesterone in *Cebpb*^ΔM^ mice as compared to WT (Fig 3D). Branch points were also quantified in these mammary glands with no significant differences (Fig 3E), suggesting that modest decreases in circulating progesterone do not alter ductal side-branching. Finally, mammary glands from *Cebpb*^ΔM^ mice exhibit normal ductal architecture, with a single layer of CK8^+^ luminal cells and a single layer of CK14^+^ myoepithelial cells (Fig S1). Together, these results suggest that deletion of C/EBPβ in macrophages reduces alveolar budding, which may be due to altered macrophage signals and moderate changes in progesterone levels.

### Macrophage deletion of *Cebpb* alters the reproductive cycle

**Figure 4.**
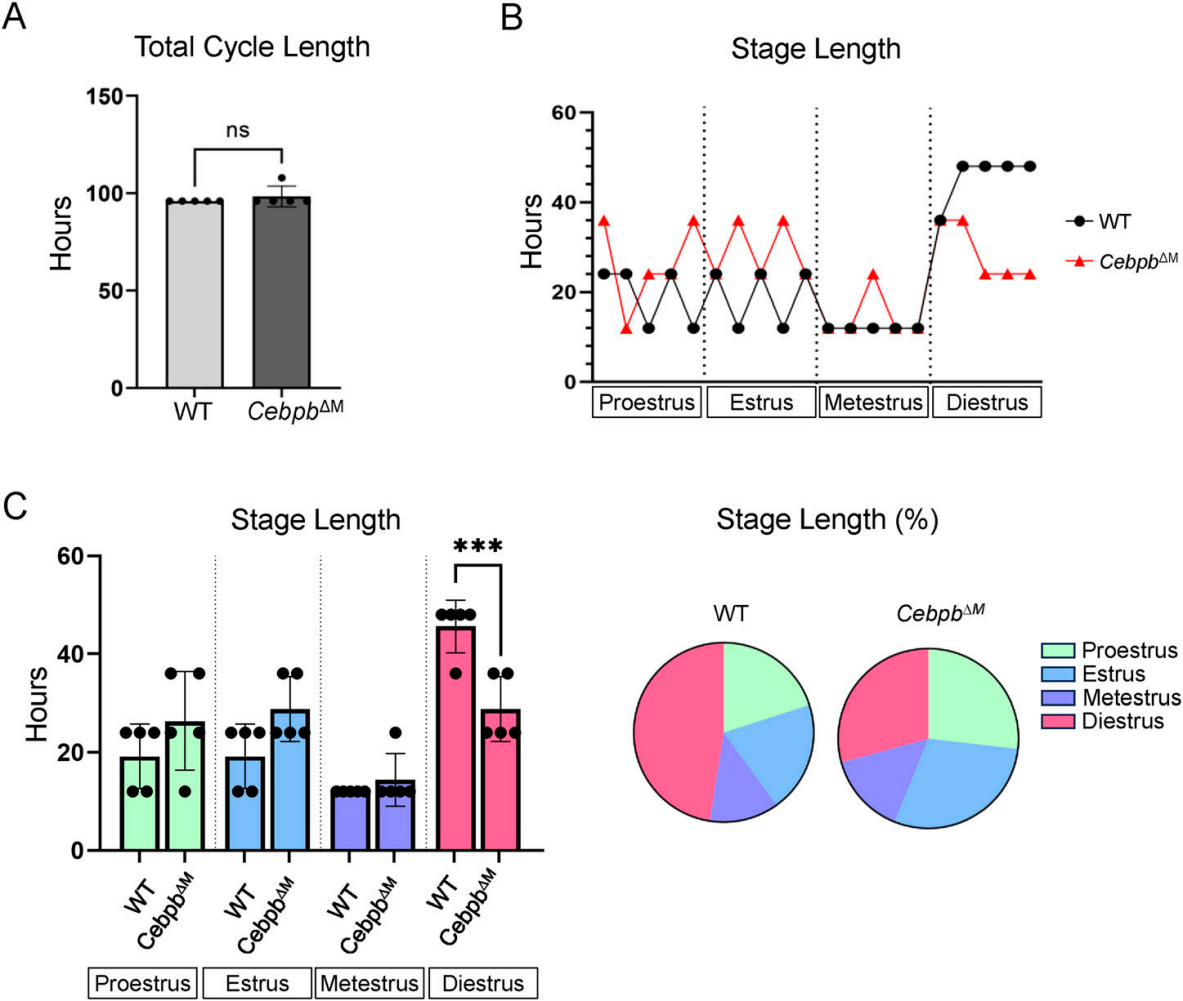
Cebpb^ΔM^ mice have a shorter diestrous stage. Reproductive cycle was tracked by vaginal cytology over a period of eight consecutive days (five mice per group, 10 wk of age). Graphs are represented in hours, which was calculated by dividing the number of days in each cycle by 2 (to represent one cycle) and then multiplied by 24 to determine the number of hours. **(A)** Graph depicts the total cycle length of one complete reproductive cycle of WT and *Cebpb*^∆M^ mice (*P* = 0.35, unpaired *t* test). **(B)** Graph shows the number of hours each individual mouse spent in each stage of the estrous cycle. **(C)** Bar graph shows the number of hours spent in each stage of the estrous cycle (***P = 0.001, one-way ANOVA with multiple comparisons). Pie charts represent the percentage of time spent in each stage of the estrous cycle.

**Figure S2.**
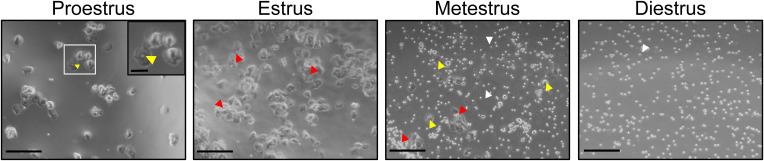
Estrous staging. Vaginal cytology was imaged with a Zeiss Axio Observer inverted microscope with phase contrast, and stages were determined by identifying nucleated or corneated epithelial cells, or leukocytes: pro-estrus, enlarged nucleated epithelial cells (yellow arrows); estrus, enlarged corneated epithelial cells (red arrows); metestrus, nucleated epithelial cells, corneated epithelial cells, and leukocytes (white arrows); diestrus, leukocytes. Scale bar = 100 μm; inset scale bar = 50 μm.

Results from E+P treatment of *Cebpb*^ΔM^ mice suggest that modest changes in systemic hormone levels contribute to reduced alveolar budding. Macrophages have also been shown to regulate ovarian physiology, and promote progesterone synthesis in the ovary (38). To address whether macrophage deletion of *Cebpb* alters ovarian function, we asked whether *Cebpb*^ΔM^ mice exhibit normal estrous cycles. Reproductive cycles were tracked by analyzing vaginal smears for a period of eight consecutive days, to ensure all stages were observed. Both WT and *Cebpb*^ΔM^ mice exhibit normal estrous cycles in that each stage is achieved for a period of time, and the overall time to complete one reproductive cycle was similar between groups (96–98 h) (Figs 4A and S2). However, *Cebpb*^ΔM^ mice undergo significantly shorter diestrus stages than WT mice, averaging approximately 30 h as compared to 48 h in WT (Fig 4B and C). These results suggest that macrophage C/EBPβ regulates ovarian function.

### Ablation of *Cebpb* in macrophages leads to the increased expression of progesterone-induced genes and pathways in the mammary epithelium

**Figure 5.**
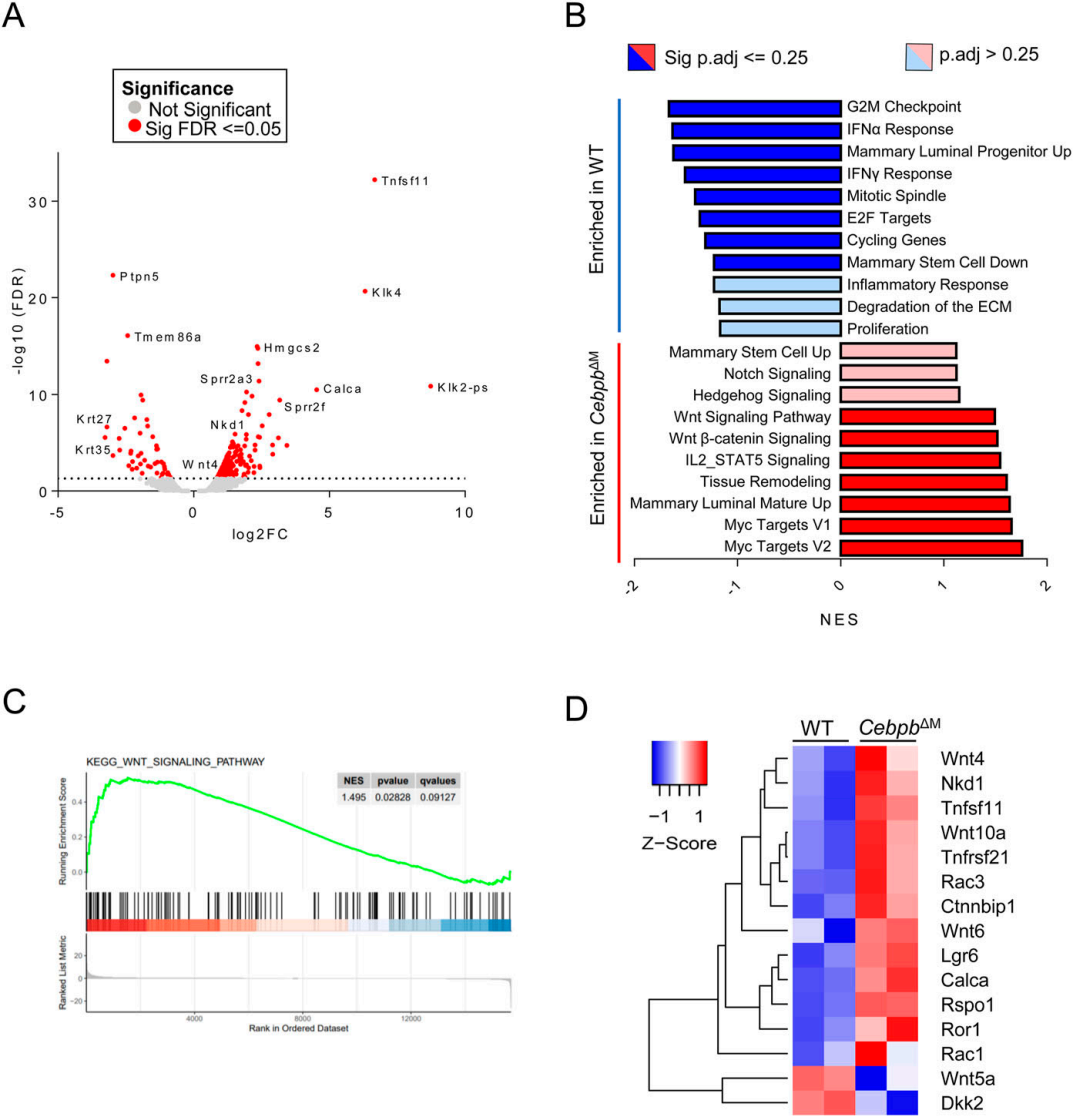
Gene expression analysis of mammary epithelial cells from *Cebpb*^ΔM^ mice. RNA sequencing was performed on mammary epithelial cells (MECs) isolated from 10-wk-old diestrus-staged WT and *Cebpb*^ΔM^ mice. **(A)** Volcano plot representation of differentially expressed genes in *Cebpb*^ΔM^ MECs as compared to WT. Red points mark the genes with significantly increased or decreased expression (*P*-adjusted <=0.05). The x-axis shows log fold changes and the y-axis the −log_10_ (FDR). **(B)** Gene set enrichment analysis identifies unique pathways enriched in WT (blue) MECs and in *Cebpb*^ΔM^ (red) MECs. Pathways were placed in order of the normalized enrichment score (*P*-adjusted <= 0.25). **(C)** Gene set enrichment analysis of the KEGG Wnt signaling pathway (*P*-adjusted = 0.12) enriched in WT MECs compared with *Cebpb*^ΔM^ MECs. **(D)** Heatmap depicts the differential gene expression of selected genes in WT and *Cebpb*^ΔM^ MECs. Genes regulated by PR or expressed in the Wnt pathway are represented, where red indicates higher expression, and blue indicates lower expression.

**Figure S3.**
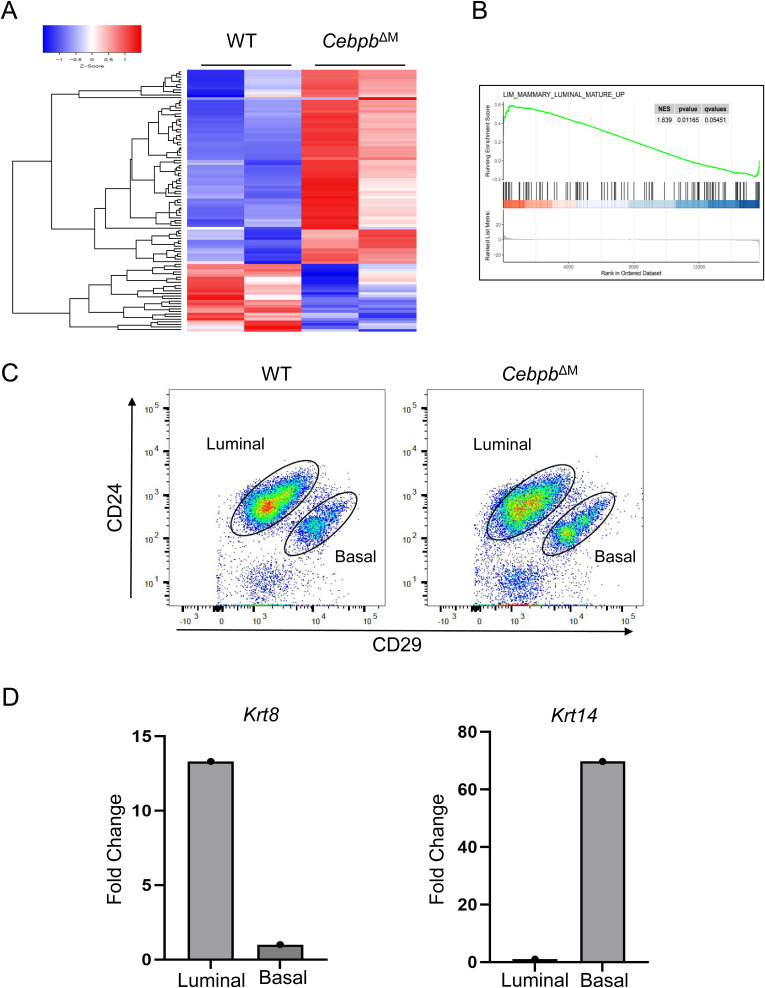
Analysis of mammary epithelial cells from Cebpb^ΔM^ mice. **(A)** Heatmap depicts the top 100 differentially expressed genes in *Cebpb*^ΔM^ MECs compared with WT, with red indicating higher expression and blue indicating lower expression. **(B)** Gene set enrichment analysis of Mammary Luminal Mature Up (*P* = 0.062) depicting WT MECs are enriched for genes expressed in mature luminal epithelial cells. **(C)** Dot plots show sorting strategy for luminal (CD45^−^CD24^+^CD29^Lo^) and basal (CD45^−^CD24^+^CD29^Hi^) cell populations from MECs isolated from the mammary glands of 10-wk-old diestrus-staged mice (three to six mice per group, with representative plots from one biological replicate). **(D)** qRT-PCR analysis of *Krt8* and *Krt14* of luminal and basal cells from WT BALB/c mice. One representative biological replicate is depicted (five mice were analyzed separately for five biological replicates).

To investigate epithelial signaling pathways that may be impacted by *Cebpb* deletion in macrophages, primary mammary epithelial cells (MECs) were isolated from diestrus-matched WT and *Cebpb*^ΔM^ mammary glands and RNA sequencing was performed. Unexpectedly, *Tnfsf11* (RANKL), *Wnt4*, and *Calca* are among the top 10 differentially expressed genes that are significantly increased in the *Cebpb*^ΔM^ epithelium as compared to WT (Figs 5A and S3A, Table S1). These three genes are known progesterone receptor (PR) target genes that mediate ductal proliferation and alveolar budding during diestrus (39). These results suggest that progesterone-induced signals that promote alveolar budding are present in *Cebpb*^ΔM^ mice. In support of these data, gene set enrichment analysis (GSEA) shows that *Cebpb*^ΔM^ MECs are enriched for pathways involving Wnt signaling as compared to WT (Fig 5B and C). Further analysis shows that numerous Wnt family members are significantly altered in *Cebpb*^ΔM^ MECs as compared to WT (Fig 5C and D, Tables S2 and S3). For example, *Wnt4*, *Wnt10a*, *Rac3*, and the Wnt antagonist *Nkd1* are significantly up-regulated in *Cebpb*^ΔM^ MECs, whereas non-canonical *Wnt5a* and Wnt agonist *Dkk2* are significantly decreased. These data suggest that ablation of C/EBPβ in macrophages results in alterations in the Wnt landscape of adjacent MECs.

Table S1. List of top differentially expressed genes.

Table S2. GSEA analysis of mammary epithelial cells.

Table S3. GSEA pathways and full GSEA names.

In addition to changes in Wnt signaling, GSEA shows that *Cebpb*^ΔM^ MECs are enriched for pathways involving Notch and Hedgehog signaling, both of which have well-established roles in stem cell function (40, 41). Interestingly, pathways involving mammary stem/progenitor cells are enriched in WT or *Cebpb*^ΔM^ MECs in a converse fashion. For example, WT MECs are enriched for the Mammary Stem Cell Down pathway, whereas *Cebpb*^ΔM^ MECs are enriched for the Mammary Stem Cell Up pathway. Likewise, WT MECs are enriched for the Mammary Luminal Progenitor UP pathway, whereas *Cebpb*^ΔM^ MECs are enriched for the Mammary Luminal Mature Up pathway (Figs 5B and S3B, Tables S2 and S3). Finally, *KlK4* and *Klk2-ps*, two peptidases that function in ECM degradation and remodeling, are also significantly increased in the *Cebpb*^ΔM^ epithelium as compared to WT, as is the GSEA pathway Tissue Remodeling (Fig 5A and B, Tables S2 and S3). Together, these data suggest that deletion of C/EBPβ in macrophages impacts important pathways that regulate tissue remodeling and stem/progenitor activity.

### *Cebpb* deletion in macrophages causes the increased numbers of PR+ cells in the adjacent epithelium

**Figure 6.**
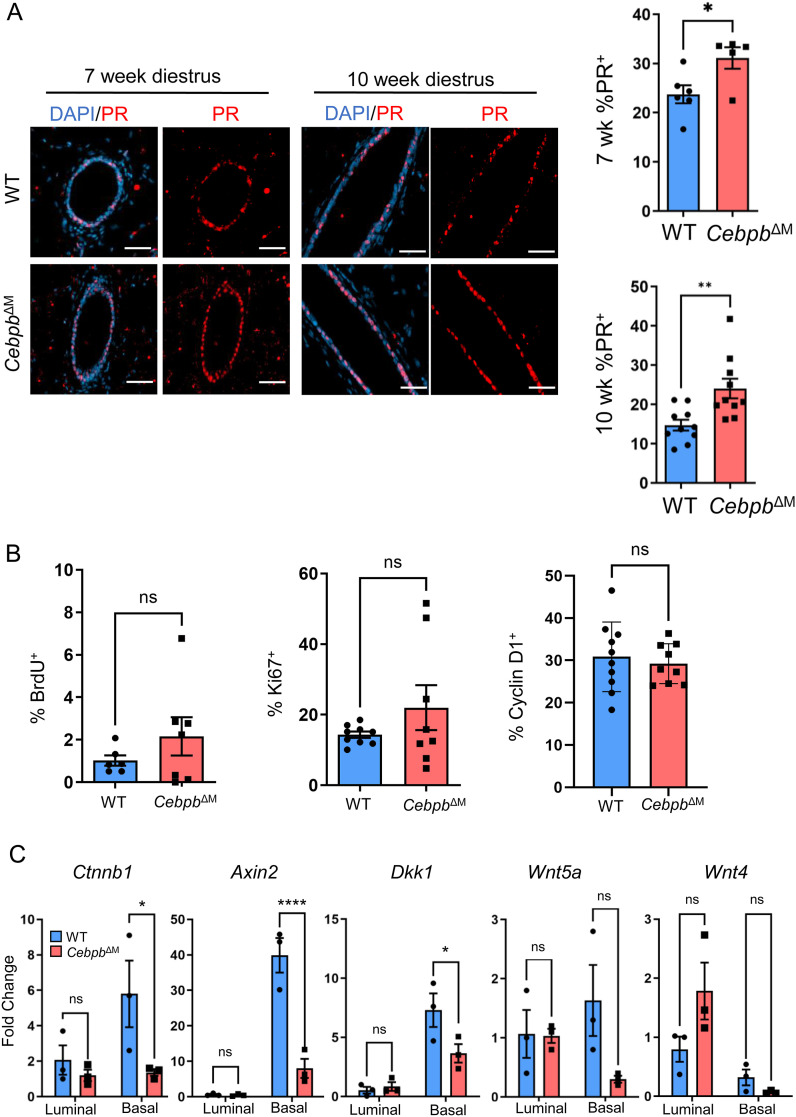
Altered expression of the PR and Wnt pathway in the *Cebpb*^ΔM^ mammary epithelium. **(A)** Representative images from 7-wk-old (20X, scale bar = 50 μm) or 10-wk-old (40X, scale bar = 40 μm) WT and *Cebpb*^ΔM^ mice harvested in diestrus, depicting PR (red) expression. Graphs show the quantification of PR^+^ mammary epithelial cells expressed as a percentage of epithelial cells. A minimum of 500 DAPI ductal cells were counted, divided by the number of PR^+^ cells, and multiplied by 100 to calculate the percent of PR^+^ cells. Values are the mean and SEM from 5 to 10 mice per group (*P = 0.03, **P = 0.004, unpaired *t* test). **(B)** Percent of ductal epithelial cells expressing BrdU (*P* = 0.28), Ki67 (*P* = 0.22), or cyclin D1 (*P* = 0.61), where a minimum of 500 DAPI ductal cells were counted, divided by the number of marker-specific positive cells, and multiplied by 100 to calculate the percent of proliferating cells. Values are shown as the mean and SEM from 6 to 10 mice per group of 10-wk-old diestrus-staged mice (unpaired *t* test). **(C)** MECs were isolated from the mammary glands of 10-wk-old diestrus-staged mice and FACS-sorted for luminal (CD45^−^CD24^+^CD29^Lo^) and basal (CD45^−^CD24^+^CD29^Hi^) epithelium. Graphs show qRT-PCR for alterations in the Wnt signaling pathway using primers to *Ctnnb1* (*P = 0.04), *Axin2* (****P < 0.0001), *Dkk1* (*P = 0.05), *Wnt5a* (*P* = 0.12), and *Wnt4* (*P* = 0.05). Values shown are the mean and SD (two-way ANOVA with multiple comparisons, three mice per group).

Previous studies have shown that progesterone signaling induces stem cell expansion and the proliferation of ductal cells during bud formation (35), where PR^+^ cells secrete Wnt4 and RANKL to stimulate the proliferation of adjacent PR^neg^ luminal cells in a paracrine fashion (42, 43). Results from E+P treatment of *Cebpb*^ΔM^ mice suggest that modest decreased systemic hormone levels contribute to reduced alveolar budding. However, branching morphogenesis was normal (Fig 3), a process that is driven by progesterone (37, 44, 45), indicating that progesterone levels are sufficient to induce side-branching. Furthermore, numerous PR target genes that induce proliferation during alveolar budding are significantly increased in the *Cebpb*^ΔM^ mammary epithelium (Fig 5). Thus, we hypothesized that local PR signaling is enhanced in *Cebpb*^ΔM^ MECs, which may lead to changes in PR-mediated Wnt signaling. To address local PR signals, PR expression and several markers of proliferation were analyzed by immunostaining WT or *Cebpb*^ΔM^ mammary glands during the diestrus phase. In support of the RNA-sequencing data, the number of PR^+^ cells is significantly increased in the epithelium of *Cebpb*^ΔM^ mammary glands as compared to WT littermates from both 7- and 10-wk-old mice (Fig 6A). Quantification of BrdU, Ki67, and cyclin D1 in MECs shows that there are no significant changes in proliferation between *Cebpb*^ΔM^ and WT mammary glands (Fig 6B). Increased PR and PR target genes accompanied by decreased budding are interesting, and suggest that the altered spatial distribution of PR^+^ cells may lead to the inability of PR^neg^ adjacent cells to receive or respond to key paracrine signals from PR^+^ cells (44, 45, 46).

### Canonical Wnt genes are decreased in *Cebpb*^ΔM^ basal cells

Our results suggest that deletion of *Cebpb* in macrophages leads to alterations in PR-responsive genes in the adjacent epithelium, without significantly affecting proliferation. PR-induced RANKL and Wnt4 have been shown to mediate mammary stem cell activity characterized by transient proliferative bursts to induce bud formation (42, 47, 48). These findings coupled with GSEA results that show alterations in stem cell pathways (Fig 5) led to the hypothesis that the basal cell component of the *Cebpb*^ΔM^ mammary epithelium, which includes stem cells, may have impaired Wnt signaling that leads to decreased mammary stem cell expansion and consequent decreased budding. In the adult mammary gland, Wnt ligands are differentially expressed in the luminal or basal cell compartments, where canonical Wnt/β-catenin signaling specifies basal cell fate and stem cell activity (49, 50). To examine canonical Wnt signaling in basal cells, primary MECs were isolated from the mammary glands of diestrus-staged WT and *Cebpb*^ΔM^ mice and FACS-sorted for LIN^−^CD24^+^CD29^Lo^ (luminal) and LIN^−^CD24^+^CD29^Hi^ (basal) populations (Fig S3C). Purity of cell populations was confirmed by qRT-PCR for *Krt8* (luminal) or *Krt14* (basal) expression (Fig S3D). In WT mammary glands, qRT-PCR analysis shows that *Ctnnb1* (β-catenin), *Axin2*, and *Dkk1* are highly expressed in basal cells as compared to luminal cells, consistent with previous reports (50, 51, 52). However, these three genes are significantly decreased in *Cebpb*^ΔM^ basal cells, indicative of decreased canonical Wnt signaling in basal cells (Fig 6C). Similar trends are shown for Wnt ligands, where *Wnt5a* mRNA is reduced in basal cells and *Wnt4* is increased in luminal cells from *Cebpb*^ΔM^ mice as compared to WT (Fig 6C). These results suggest that ablation of C/EBPβ in macrophages results in decreased canonical Wnt signaling in adjacent basal cells, which normally promotes stem cell expansion during bud formation.

### C/EBPβ-deficient macrophages exhibit increased phagocytic activity and down-regulate *Notch*

**Figure 7.**
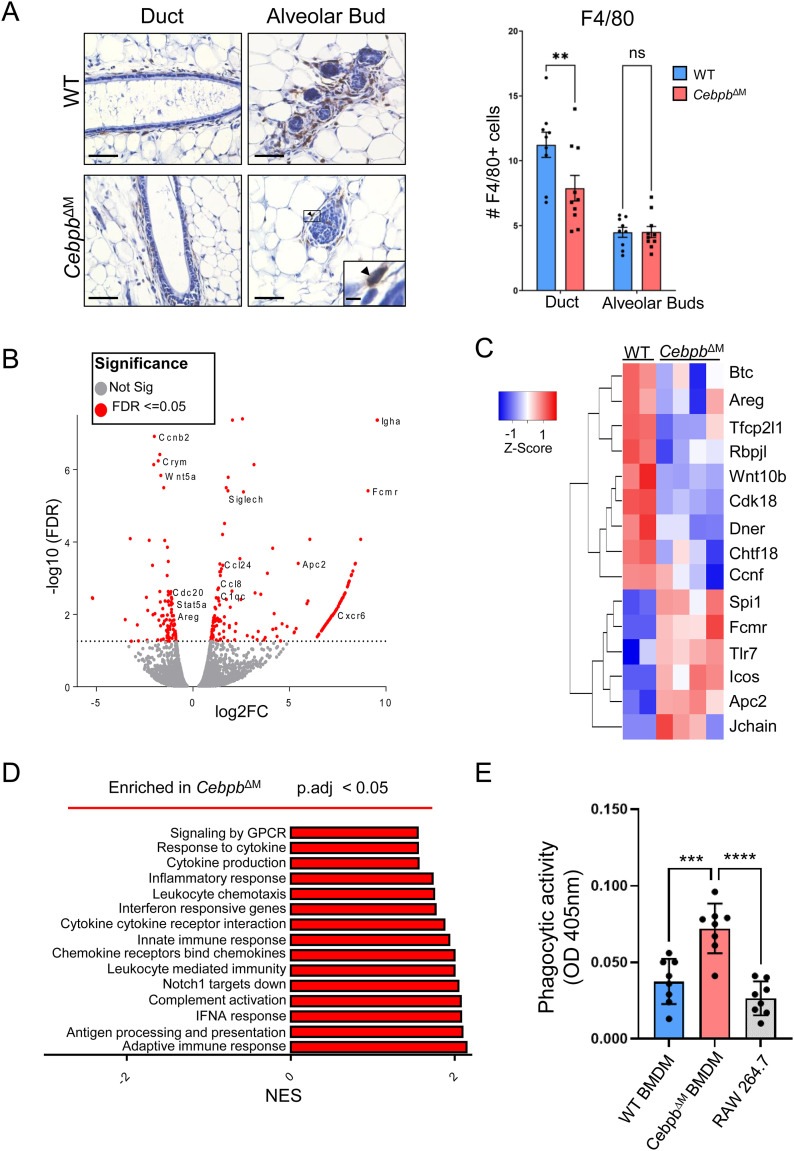
Altered gene expression and function in *Cebpb*^ΔM^ macrophages. **(A)** Representative 40X images (inset 200X) of F4/80 immunostaining in 10-wk-old mammary glands from WT and *Cebpb*^ΔM^ diestrus-staged mice (five mice per group) (scale bar = 40 μm; inset scale bar = 0.2 μm; arrow depicts F4/80+ cell). The graph shows the quantitation of the average number of F4/80+ cells per FOV. A minimum of five FOV (at least five ductal or five alveolar buds) were counted for each group (**P = 0.006, two-way ANOVA with multiple comparisons). **(B)** Volcano plot representation of differentially expressed genes in *Cebpb*^ΔM^ macrophages as compared to WT. Red points mark the genes with significantly increased or decreased expression (−log_10_[FDR] > 2). **(C)** Heatmap depicts differentially expressed genes in *Cebpb*^ΔM^ macrophages compared with WT. **(D)** GSEAs showing pathways enriched in *Cebpb*^ΔM^ macrophages. Pathways were placed in order of the normalized enrichment score (*P*-adjusted <0.05). **(E)** Phagocytic activity of BMDMs from WT and *Cebpb*^ΔM^ mice measured by absorbance (OD 405 nm) using Phagocytosis Assay Kit, where *Cebpb*^ΔM^ BMDMs have increased ability to phagocytose Zymosan particles. RAW 264.7 cells serve as a positive control. Values are the mean and SEM (***P = 0.0002, ****P < 0.0001, one-way ANOVA with multiple comparisons). Eight technical replicates are shown for one representative experiment, and the experiment was performed three times (three biological replicates).

Although progesterone induces epithelial-derived proliferation signals, macrophages also secrete factors that act on adjacent basal cells to stimulate mammary stem cell expansion. To address whether the observed changes in the *Cebpb*^ΔM^ epithelium are the result of decreased macrophage recruitment, F4/80^+^ cells were quantified in the adjacent epithelium. Although there is a reduction in the number of macrophages being recruited to the ductal epithelium of *Cebpb*^ΔM^ mammary glands as compared to WT, the number of macrophages recruited to alveolar buds or the distal tips remains unchanged (Fig 7A). These data suggest that budding alterations are likely not due to decreased macrophage numbers.

**Figure S4.**
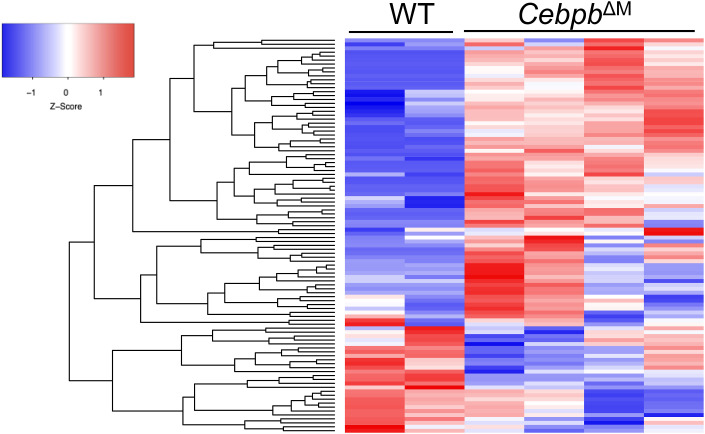
Differentially expressed genes in *Cebpb*^ΔM^ macrophages. Heatmap depicts the top 100 differentially expressed genes in *Cebpb*^ΔM^ macrophages compared with WT, with red indicating higher expression and blue indicating lower expression.

To identify C/EBPβ-induced macrophage factors that contribute to alveolar budding of adjacent epithelial cells, RNA sequencing was performed on macrophages. Immune cells were isolated from the mammary glands of WT and *Cebpb*^ΔM^ mice, and macrophages were enriched using an F4/80 positive selection kit. Analysis of *Cebpb*^ΔM^ macrophages shows 263 differentially expressed genes (*P*-adjusted <0.05), with 84 genes down-regulated and 179 genes up-regulated when compared to WT macrophages (Figs 7B and S4, Table S1). Differentially expressed genes that are significantly increased in *Cebpb*^ΔM^ macrophages contribute to chemotactic, inflammatory functions (*Cxcr6*, *Ccl8*, *Ccl24*) and complement activation (*C1qc*). Immunoregulatory and phagocytic factors (*Tlr7*, *Icos*, *Fcmr*, *Siglech*, *Lamp5*) and macrophage activation genes (*Jchain*, *Spi1*) are also up-regulated (Fig 7C, Table S1). Similarly, GSEA shows that *Cebpb*^ΔM^ macrophages are significantly enriched for various immune response pathways, which may suggest that *Cebpb*^ΔM^ macrophages are more immunologically active (Fig 7D, Tables S3 and S4). It should be noted that several epithelial factors (*Krt14*, *Csn2*, *Csn1s1*) are also enriched in C/EBPβ-deficient macrophages, which may be indicative of phagocytosed epithelial cells. These results led to the hypothesis that *Cebpb*^ΔM^ macrophages have increased phagocytic activity as compared to WT. To test this hypothesis, BMDMs were isolated from WT or *Cebpb*^ΔM^ mice and assessed for their ability to engulf Zymosan particles. Compared with WT cells, C/EBPβ-deficient BMDMs have a significant increase in phagocytic capacity (Fig 7E), supporting a more immunologically active phenotype.

Table S4. GSEA analysis of macrophages.

**Figure 8.**
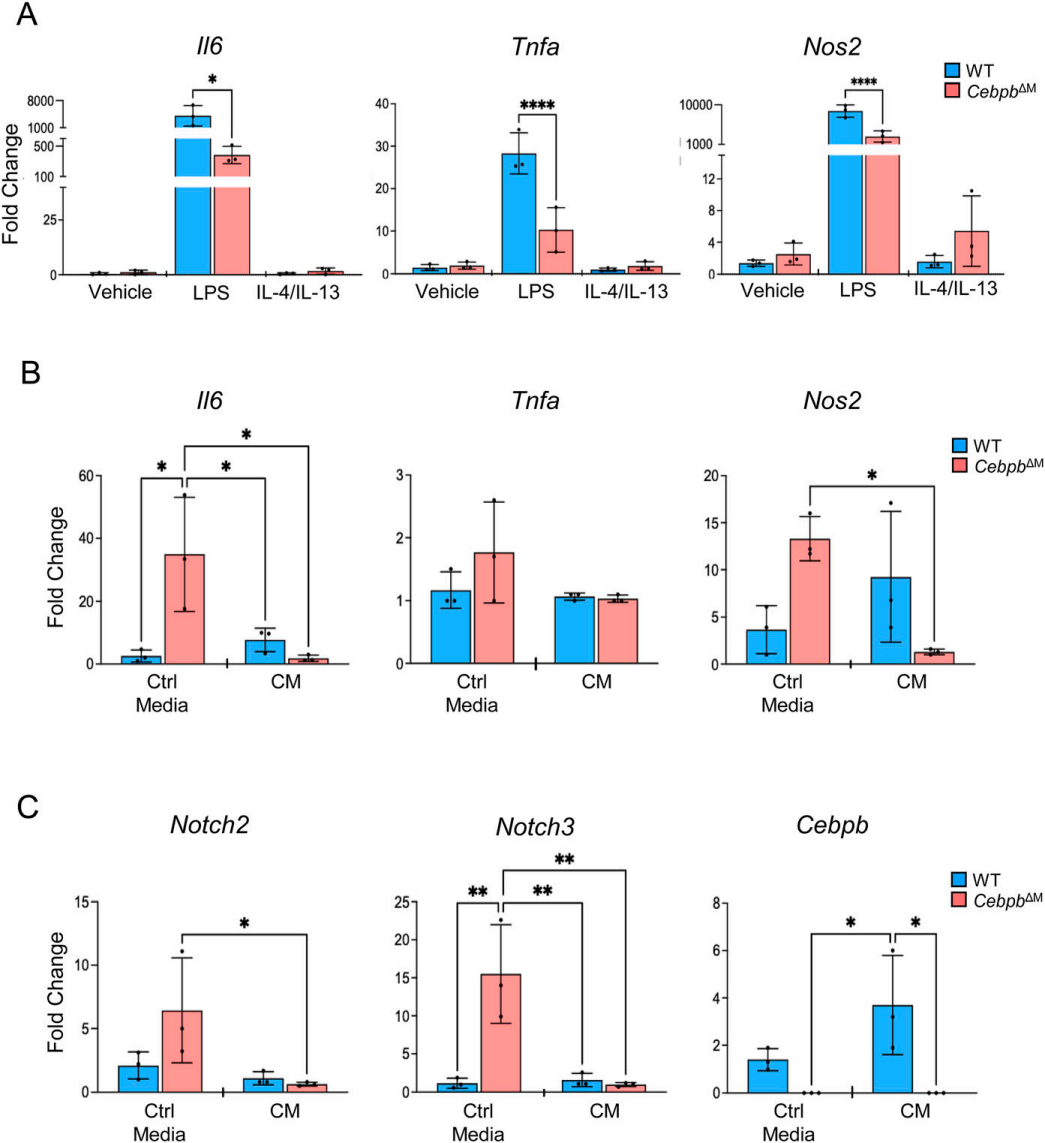
Cebpb^ΔM^ macrophages have the reduced expression of pro-inflammatory cytokines and Notch. **(A)** BMDMs from WT and *Cebpb*^ΔM^ mice (three mice per group) were treated with vehicle, LPS (4 μg/ml), or IL-4 (20 ng/ml) and IL-13 (20 ng/ml) for 24 h, and qRT-PCR was performed using primers to *Il6* (*P = 0.01), *Tnfa* (****P < 0.0001), and *Nos2* (****P < 0.0001). **(B, C)** BMDMs from WT and *Cebpb*^ΔM^ mice (three mice per group) were treated with control (unconditioned) media or HC11 conditioned media for 72 h, and qRT-PCR was performed using primers to *Il6* (*P < 0.03), *Tnfa*, *Nos2* (*P = 0.02), *Notch2* (*P = 0.04), *Notch3* (**P < 0.004), and *Cebpb* (*P = 0.01). For all data, the mean and SD are represented (one-way ANOVA with multiple comparisons).

Because high phagocytic capacity has been reported in anti-inflammatory, tissue-reparative macrophages (53, 54), we asked whether C/EBPβ-deficient macrophages demonstrate a shift in the macrophage phenotype. WT or *Cebpb*^ΔM^ BMDMs were treated with LPS to induce a pro-inflammatory phenotype (M1-like), or IL-4/IL-13 to induce an anti-inflammatory phenotype (M2-like), and various cytokines were analyzed by qRT-PCR. *Cebpb*^ΔM^ BMDMs show a significant decrease in *Il6*, *Tnfa*, and *Nos2* in LPS-treated macrophages as compared to WT, suggesting the reduced expression of pro-inflammatory cytokines (Fig 8A). Likewise, BMDMs were treated with control (unconditioned) or conditioned media from the MEC line HC11 to determine whether epithelial-derived factors influence the macrophage phenotype. Notably, control media induce *Il6* and *Nos2* in *Cebpb*^ΔM^ BMDMs as compared to WT. However, *Cebpb*^ΔM^ BMDMs significantly decrease the expression of these cytokines when treated with HC11 conditioned media as compared to control media, suggesting an anti-inflammatory macrophage phenotype. *Tnfa* expression was barely detectable (i.e., CT > 38) in all groups analyzed (Fig 8B). Together, these data suggest that *Cebpb*^ΔM^ macrophages are highly phagocytic and reduce the expression of pro-inflammatory cytokines, suggesting the adoption of an M2-like tissue-reparative phenotype, consistent with enhanced tissue remodeling during alveolar budding.

Macrophages also secrete Wnt ligands during alveolar budding. Previous studies showed that macrophages and epithelial cells cooperatively induce Wnt ligands above a threshold to promote proliferation and mammary stem cell expansion. Specifically, macrophage-derived Notch2 and Notch3 induce Wnt ligand production in macrophages, which interact with basal cells to induce mammary stem cell expansion (55). RNA sequencing shows that down-regulated genes in *Cebpb*^ΔM^ macrophages include those involved in cell cycle regulation (*Ccnb2*, *Ccnf*, *Cdk18*, *Chtf18*), and Wnt molecules (*Wnt5a*, *Wnt10b*, *Wnt4*, *Wnt2*). Likewise, mediators of Wnt signaling (*Tfcp2l1*) and Notch signaling (*Rbpjl, Dner*) are down-regulated in *Cebpb*^ΔM^ macrophages, whereas the Wnt pathway inhibitor, *Apc2*, is up-regulated (Fig 7C, Table S1). Although these data are interesting, they should be cautiously interpreted, as it is unclear as to whether these genes are expressed by the macrophages or by phagocytosed epithelial cells. To avoid misinterpretation of results, BMDMs treated with conditioned media from HC11 cells were analyzed. HC11 cells induce *Cebpb* expression in a similar fashion to LPS, suggesting that MECs secrete factors that promote an M1-like phenotype in this system. Interestingly, control media induce both Notch2 and Notch3 in *Cebpb*^ΔM^ BMDMs; however, conditioned media significantly decrease these genes in *Cebpb*^ΔM^ macrophages (Fig 8C). These results suggest that C/EBPβ regulates Notch expression in macrophages, providing a potential link between macrophage C/EBPβ and alveolar budding.

## Discussion

Macrophages have critical homeostatic functions that balance organ health, and disruption of tissue homeostasis can lead to disease. Genetic ablation studies and other approaches have demonstrated that macrophages play a central role in regulating mammary gland development and breast cancer progression (4, 5, 6, 7, 56, 57, 58). However, the macrophage-derived factors that contribute to mammary gland tissue homeostasis are not fully understood. In the present study, we sought to identify key macrophage factors and signaling pathways important for normal mammary gland function. Our results highlight the importance of macrophage signaling in maintaining normal epithelial morphology during the estrous cycle, with C/EBPβ as a novel driver of macrophage function in the mammary gland.

C/EBPβ is expressed in numerous cell types and is canonically known to regulate the balance between proliferation and differentiation (23, 59, 60, 61). In immune cells, C/EBPβ modulates inflammatory and anti-inflammatory cytokine production, and thus is an important factor in regulating the immune response (26, 28, 62). Given the ubiquitous expression of C/EBPβ in various subpopulations of CSF1R-expressing myeloid cells in both the normal mammary gland (Fig 1) and a mouse model of breast cancer progression (32), we hypothesized that macrophage-derived C/EBPβ may be fundamental to macrophage function in the mammary gland. To address this question, a conditional knockout strategy was developed where C/EBPβ is genetically deleted from CSF1R^+^ macrophages. Although ductal morphogenesis remained normal, there was a significant decrease in alveolar budding and remodeling of the stroma during diestrus (Fig 3), a period of the estrous cycle that is characterized by transient proliferative bursts of the epithelium. These alterations were associated with increased levels of PR target genes (Figs 5 and 6). Previous studies using germline knockout mice have shown that *Cebpb*^*−/−*^ mice have decreased ovarian steroid hormone production, abnormal ductal morphogenesis, and decreased lobuloalveolar differentiation during pregnancy. Elegant transplant studies showed that these alterations were due to being epithelial-intrinsic, rather than due to a lack of systemic hormone production (23). However, our studies show that *Cebpb*^ΔM^ mice exhibit a shorter diestrus stage length than WT mice, with a trend of decreased circulating progesterone (Figs 3 and 4). Treatment with exogenous hormones (E+P) partially rescued the observed alveolar defects, which suggests that moderate changes in progesterone can affect alveolar budding (Fig 3). *Cebpb*^*−/−*^ mammary epithelium also exhibits a block in the cell cycle resulting in decreased proliferation, and altered PR expression in nulliparous mice stimulated with progesterone and estradiol to mimic pregnancy (46). Although macrophage deletion of C/EBPβ did not affect epithelial proliferation, similar results in the present study were obtained showing deficient alveolar budding and an increased number of PR^+^ cells in the adjacent epithelium (Fig 6). These data suggest that macrophages may contribute, at least partially, to the previously observed phenotypes in *Cebpb*^*−/−*^ mice, and highlight the importance of crosstalk between immune cells and the epithelium during mammary gland development. Future studies will continue to investigate the macrophage C/EBPβ function at different stages of mammary gland development, such as pregnancy and involution, where macrophages play important roles in tissue remodeling.

Systemic hormones are crucial for proper mammary gland development, particularly during ductal morphogenesis, the estrous cycle, and pregnancy (63, 64). For example, estradiol and progesterone are necessary for ductal elongation and branching morphogenesis during puberty, respectively, and prolactin is critical for milk production during pregnancy and lactation (65, 66, 67). During diestrus, progesterone has well-established roles in mediating alveolar budding. Ovariectomized adult mice fail to form alveolar buds, which can be rescued through exogenous estradiol and progesterone (34, 66). In addition, PR deletion in MECs results in decreased side-branching in mammary glands from pubertal, adult, and pregnant mice (44, 68, 69). Although PR alterations have been linked to deficient side-branching (59), branching morphogenesis in *Cebpb*^ΔM^ mammary glands was normal (Fig 3). Similar to these findings, macrophage depletion during diestrus has been shown to result in reduced numbers of alveolar buds without affecting branching. Macrophages were shown to promote the development of alveolar buds in preparation for pregnancy, and subsequently remodel the tissue by inducing bud regression to prepare for a new estrous cycle (2). Our results suggest that C/EBPβ is important for macrophage-directed bud formation during the reproductive cycle.

During diestrus, there are transient proliferative bursts with increased stem cell activity, regulated by progesterone-induced RANKL and Wnt4 (42, 47, 48). The increase in the number of PR^+^ cells in the *Cebpb*^ΔM^ mammary epithelium that was accompanied by significant increases in *Tnfsf11* (RANKL) and *Wnt4* (Fig 5) suggests a unique mechanism by which macrophages contribute to local PR signaling. Furthermore, genes indicative of canonical Wnt signaling (*Ctnnb1* [β-catenin], *Axin2*) were significantly decreased in the basal cells of *Cebpb*^ΔM^ mammary glands (Fig 6). Both canonical (β-catenin–dependent) and non-canonical (β-catenin–independent) Wnt signaling have been well studied during mammary gland development, where both pathways play important roles in pubertal morphogenesis and in adult mammary epithelium maintenance (reviewed in reference 39). For example, Ror2, the receptor for Wnt5a/Wnt5b, orchestrates branching morphogenesis in a Wnt/β-catenin–independent fashion. Disruption of Ror2 in MECs leads to changes in Wnt ligand expression in the luminal and basal cell compartments, resulting in alterations in cytoskeletal dynamics (70). Wnt4 is induced by estradiol and progesterone, and is predominantly expressed in PR^+^ luminal cells. Studies from *Wnt4*^*−/−*^ mice, as well as in vitro culture and in vivo transplantation assays, showed that Wnt4 regulates the expansion of basal mammary stem cells, and reduces the regenerative capacity of these cells in vivo (48, 49, 71). Our data are consistent with the latter, and suggest that ablation of C/EBPβ in macrophages leads to changes in the composition of MECs, potentially altering the Wnt landscape during a key window susceptible to transient expansion of stem cells. It is tempting to speculate that macrophage C/EBPβ coordinates stem cell expansion during alveolar budding; however, further studies are required.

Several studies have provided evidence that macrophages are necessary for mammary stem cell function, and depletion of macrophages results in impaired ductal outgrowth, a process that requires regenerative stem cell activity (2, 5, 7). More recently, Dll1 produced by mammary stem cells was shown to activate Notch in macrophages, which in turn induces Wnt ligands that stimulate stem cell expansion (55). These studies highlight the close interactions between mammary stem cells and adjacent macrophages, and demonstrate the importance of Wnt signaling in both macrophages and epithelial cells of the mammary gland. Extensive studies have shown that macrophages are a source of Wnt ligands and that Wnt signaling regulates macrophage function. Interestingly, there was a decrease in *Wnt4*, *Wnt5a*, and *Wnt10b* in *Cebpb*^ΔM^ macrophages as compared to WT, whereas the Wnt inhibitors *Wif1* and *Amer2* were increased (Fig S4). The GSEA pathway Notch 1 Targets Down was enriched in *Cebpb*^ΔM^ macrophages (Fig 7), providing further evidence that ablation of C/EBPβ in macrophages disrupts macrophage–epithelial crosstalk, potentially impairing stem cell expansion. In addition, *Cebpb*^ΔM^ macrophages were enriched for pathways involving the inflammatory response, and showed increased phagocytic activity and decreased expression of pro-inflammatory cytokines (Figs 7 and 8). We propose that loss of C/EBPβ alters the biology of macrophages where a tissue-reparative phenotype is adopted, allowing for premature bud regression during diestrus. Alternatively, alveolar budding may be absent in *Cebpb*^ΔM^ mammary glands, although further studies are required to answer these questions. In conclusion, our results show that deletion of C/EBPβ in macrophages leads to alterations in the reproductive cycle, disrupted PR-mediated responses, and enhanced macrophage activity. These studies provide important insights into the mechanisms by which macrophages direct bud remodeling and maintain tissue homeostasis in the mammary gland.

## Materials and Methods

### Animal models

Mice were housed in a pathogen-free facility as recommended by the NIH Guide for the Care and Use of Experimental Animals. All animal care and procedures were approved by the Tulane School of Medicine Institutional Animal Care and Use Committee and were in accordance with the procedures detailed in the Guide for Care and Use of Laboratory Animals. *Cebpb-floxed* mice were provided by Dr. Esta Sterneck (NCI, Frederick), Rosa^mTmG^ mice were provided by Dr. Michael Lewis (Baylor College of Medicine, #007676; Jax), and both lines were backcrossed 10 generations to the BALB/c background. *Csf1r-iCre* mice were purchased from Jackson Laboratories (#021024; Jax) and backcrossed to the BALB/c background using the speed congenic technology provided by IDEXX RADIL (72). BALB/c mice were obtained from Envigo (BALB/cAnNHsd). For conditional deletion of C/EBPβ, *Csf1r-Cre*^*neg*^*;Cebpb*^*fl/fl*^ mice were used as WT controls, and *Csf1r-Cre*^*pos*^*;Cebpb*^*fl/fl*^ mice served as mutant mice (*Cebpb*^ΔM^), unless indicated otherwise.

### Estrous staging and hormone treatment

Vaginal lavages were collected from 10-wk-old female mice at the same time of day for a period of eight consecutive days to ensure each stage was achieved at least twice, and prepared for staging as previously described (73). Vaginal cytology was imaged with a Zeiss Axio Observer inverted microscope, and stages were determined by identifying nucleated or corneated epithelial cells, or leukocytes (73). The number of days each stage occurred was divided by 2 to represent one cycle, and then multiplied by 24 to calculate the number of hours each stage occurred. For hormone treatment (E+P), age-matched adult female mice were injected s.c. with 1 μg β-estradiol and 1 mg progesterone (Thermo Fisher Scientific) in sesame oil once daily for 4 d (74).

### Single-cell RNA-seq data analysis

The single-cell RNA-sequencing analysis of immune cells from 10-wk-old diestrus FVB/NJ mice was previously published (30), and sequencing data were deposited in GEO under the accession code GSE148209. The gene expression values were generated by the 10X Genomics Cell Ranger, and downstream processing was performed using Seurat v3.1.1. High-dimensional gene expression profiles were reduced to a two-dimensional representation using the t-SNE (t-distributed stochastic neighbor embedding) projection. Cells were then colored by the normalized expression of the genes in the cell.

### Immunostaining

Paraffin-embedded mammary glands were sectioned at 5 μm, deparaffinized, rehydrated, and stained with hematoxylin and eosin. F4/80 staining was performed in the absence of antigen retrieval as previously described (75). Peroxidases were quenched with 3% H_2_O_2_ in methanol, blocked with M.O.M. blocking reagent (Vector Laboratories), and incubated with antibodies overnight at 4°C. Antibodies and dilutions are listed in Table S5. The next day, slides were washed with PBS and incubated with a biotinylated antibody (1:500) (Vector Laboratories) for 30 min. Slides were washed with PBS, incubated for 10 min with the VECTASTAIN Elite ABC-HRP reagent, R.T.U (Vector Laboratories), and developed using a DAB peroxidase substrate kit (Vector Laboratories). Glands were counterstained with hematoxylin, dehydrated, and mounted with Permount (Thermo Fisher Scientific). For quantification of macrophages, 5 FOV (at least five ducts and five alveolar buds) from each gland were counted from five mice.

Table S5. List of antibodies.

For immunofluorescence, tissues were blocked with 3% BSA and stained with antibodies (76) (Table S5). The next day, slides were washed with PBS and stained with Alexa Fluor–conjugated secondary antibodies (Thermo Fisher Scientific) for 2 h at RT. Slides were mounted with ProLong Diamond Antifade Mountant containing DAPI (Thermo Fisher Scientific). Images were acquired using a Zeiss microscope (Axio Imager.A2) under a 20X or 40X objective. For quantification of PR, BrdU, Ki67, and cyclin D1, a minimum of 500 DAPI cells were counted and divided by the number of positive cells, which was calculated as a percentage.

### Whole-gland immunofluorescence

Mammary glands were harvested and fixed in 4% PFA for 2 h. Mammary glands were optically cleared using the SeeDB-based clearing method as previously described (77). Briefly, mammary glands were blocked and permeabilized overnight at 4°C in PBS with Triton X-100 (1% [wt/vol]) and BSA (10% [wt/vol]). Tissue was incubated with DAPI (10 μM) for 3 h at RT with gentle rocking in blocking buffer. Samples were serially incubated for 8–16 h in 2–3 ml of 20%, 40%, 60%, and 80% fructose in distilled water, and 100% and 115% fructose (wt/vol) for 24 h. All fructose solutions contained α-thioglycerol (0.5% [vol/vol]), and incubations were performed with gentle rocking. Images were obtained with Nikon Eclipse Ti2 Confocal Microscope under a 40X objective.

### Isolation and treatment of BMDMs

Bone marrow was obtained from the femur and tibia of BALB/c mice as previously described (78). Briefly, 10 × 10^6^ bone marrow cells were cultured in a 10-cm petri dish for one week in DMEM (Life Technologies) supplemented with 10% FBS (Life Technologies), 1% penicillin and streptomycin (Life Technologies), and 20% L929 conditioned media (79). After 7 days of culture, BMDMs were dissociated from the dish and 0.5 × 10^6^ cells were plated in 6-well plates. Cells were treated with vehicle, LPS (4 μg/ml), or IL-4 (20 ng/ml) and IL-13 (20 ng/ml) overnight and then harvested for protein or mRNA analyses. For conditioned medium treatment, HC11 cells were cultured in RPMI 1640 growth media (10% FBS, 10 ng/ml EGF, 5 μg/ml insulin) for 24 h, when conditioned or control media (unconditioned) were added to BMDMs that had been serum-starved overnight. After 18 h, cells were harvested for mRNA isolation. All cell lines were authenticated by STR Profiling (DDC Medical).

### Immunoblotting

Cells were lysed in RIPA buffer containing protease and phosphatase inhibitors. Lysates were centrifuged at 14,000*g* at 4°C for 10 min. Protein concentration was quantified using Pierce BCA Protein Assay Kit according to the manufacturer’s instructions. Equal amounts of protein (100 μg) were separated by a 10% SDS–PAGE. Proteins were transferred to a polyvinylidene difluoride membrane (Bio-Rad) for 1 h at 100 V. Membranes were blocked with 5% skim milk in TBST. Specific proteins were detected by incubating membranes overnight with antibodies listed in Table S5. Then, membranes were washed and incubated with HRP-conjugated secondary antibodies (dilution 1:5,000) for 1 h. Bands were detected using ECL Western Blotting Substrate.

### RNA isolation and quantitative RT–PCR

Total RNA was extracted from RAW 264.7 cells (ATCC), primary BMDMs, or MECs using TRIzol according to the manufacturer’s instructions. Contaminating DNA was removed using DNA-free DNA Removal Kit (Thermo Fisher Scientific), and cDNA was prepared using iScript cDNA Synthesis Kit (Bio-Rad) according to the manufacturer’s instructions. qRT-PCR was performed using SYBR Green methodology on CFX96 Touch Real-Time Detection System (Bio-Rad). The primer sequences are listed in Table S6, and the relative gene expression changes were determined using the 2-ΔΔCt method, where 18S served as the internal control. For each gene, at least three biological replicates were analyzed.

Table S6. Primer sequences for qRT-PCR.

### Whole-mount analysis

For whole-mount analysis, inguinal (#4) mammary glands were harvested from 5-, 7-, and 10-wk-old virgin female mice (5–7 mice per timepoint). The glands were fixed in Carnoy’s fixative for 2 h and stained with carmine alum overnight. Glands were dehydrated in a series of ethanols and placed in xylene before imaging on a Leica M165 FC stereoscope (Leica Biosystems). Ductal elongation was quantified with NIS-Elements Basic Research Software (Nikon Instruments) by measuring the distance (mm) from the center of the lymph node to the last end bud or terminal ductal unit. Branching morphogenesis and alveolar budding were quantified by counting all respective structures in the entire mammary gland. The number of TEBs in the entire mammary gland was quantified.

### ELISA

Blood was collected from 10-wk-old diestrus-staged BALB/c females via heart puncture. Whole blood was transferred into tubes with anticoagulant and centrifuged at 2,000*g* for 15 min at RT. Plasma was collected and stored at −20°C. Progesterone (IBL) and estradiol (Calbiotech) ELISAs were performed according to the manufacturer’s instructions.

### MEC isolation

Mammary glands (#3, 4, and 5) were dissected from 10-wk-old diestrus-staged BALB/c mice, minced, and digested for 1 h in a DMEM/F12 (Life Technologies) medium containing 2 mg/ml of collagenase A (Roche) and 1% antibiotic–antimycotic (Life Technologies). Lymph nodes were removed from all mammary glands before mincing. Digestion was neutralized by adding DMEM/F12 (Life Technologies), supplemented with 10% FBS (Life Technologies) (75). Differential centrifugation separated epithelial organoids, 0.05% trypsin–EDTA dissociated cells to a single-cell solution, and cells were passed through a 70-μm filter. Cells were purified using a LIN negative selection EasySep kit (#19868; StemCell Technologies).

### Mammary gland macrophage isolation

Macrophages were isolated by harvesting the #3, 4, and 5 mammary glands, removing the lymph nodes, and mincing the tissue. Tissue was digested for 45 min in a DMEM/F12 (Life Technologies) medium containing 1 mg/ml of collagenase A (Roche) and 1% antibiotic–antimycotic (Life Technologies). Digestion was neutralized by adding DMEM/F12 (Life Technologies), supplemented with 10% FBS (Life Technologies). Cells were filtered through a 70-μm cell strainer and treated with ACK lysis buffer. Cells were purified using an F4/80 positive selection kit (Thermo Fisher Scientific).

### Bulk RNA sequencing and analysis

MECs or mammary gland macrophages were isolated as described above from 10-week-old WT and *Cebpb*^ΔM^ female mice in the diestrus stage. RNA was prepared using TRIzol reagent as described above and submitted to BGI for RNA sequencing. Low input RNA-seq was performed on the DNB-seq platform, with paired-end 150-bp, 20 million reads per sample, where raw FASTQ files were generated. Analyses of differentially expressed genes in the MEC and macrophage samples were performed at the Minnesota Supercomputing Institute at the University of Minnesota. Briefly, FASTQ files were first processed with the CHURP pipeline (80) to perform adaptor trimming using Trimmomatic (version 0.33) (81); reads were then mapped to GRCm38 mouse genome using HiSat2 (version 2.1.0) (82). The subread count was generated using the Subread featureCounts tool (version 1.6.2) (83) using the Mus_musculus.GRCm38.100.gtf annotation. Count data were filtered by removing genes that were less than 300 nt in length and had a cpm (counts per million) value greater than 1 cpm in at least two sample replicates. The likelihood ratio test was used to evaluate differential expression (DE) with edgeR (version 3.38.1) (84, 85). The Benjamini–Hochberg method was used to adjust *P*-values for multiple hypothesis testing, and an adjusted *P*-value ≤ 0.05 with a log_2_ fold change > 0 was used as a DE significance threshold. For gene ontology (GO) pathway and GSEA, the R package clusterProfiler (version 4.4.4) (86, 87) was used. Specifically, for MEC RNA-seq GSEA, Hallmark and 13 selected pathways were combined and used for testing; for macrophage RNA-seq GSEA, all GSEA C2 canonical pathways (KEGG, GOBP, GOCC, GOMF) were tested by individual pathway sets. Full GSEA names are described in Table S3.

### FACS

MECs were harvested and purified from 10-wk-old mice in the diestrus stage as described above. Cells were incubated on ice for 30 min with fluorophore-conjugated anti-mouse antibodies as indicated in Table S5. Subsequently, cells were washed with HBSS (Life Technologies) containing 2% FBS (Life Technologies), filtered through a 35-μm cell strainer (BD Bioscience), and stained with 5 nM SYTOX Red. Cells were sorted using a FACSAria IIu cell sorter.

### Phagocytosis assay

BMDMs or RAW 264.7 (positive control) cells were seeded at 1 × 10^4^ cells/well of a 96-well plate and incubated at 37°C overnight to allow for attachment. Cells were subjected to Phagocytosis Assay Kit (Zymosan Substrate) according to the manufacturer’s protocol (ab211156; Abcam). Phagocytic activity was quantified by measuring absorbance at 405 nm of engulfed Zymosan particles.

### Statistical analysis

All statistical analyses were performed using GraphPad Prism 10. Western blots are representative of at least three independent experiments. For single comparisons, the significance was calculated using an unpaired *t* test, as indicated in the figure legends. For two or more categorical variables, either two-way ANOVA or one-way ANOVA with Sidak’s or Tukey’s multiple comparison test was performed, as indicated in the figure legends. For multiple comparisons, all *P*-values are shown in Table S7.

Table S7. *P*-values for multiple comparisons tests.

## Supplementary Material

Reviewer comments

## Data Availability

RNA-seq data generated in this study have been deposited to Gene Expression Omnibus (GEO) and can be found under the accession number GSE225295.
