# Supplementary material for: C/EBPβ deletion in macrophages impairs mammary gland alveolar budding during the estrous cycle
Source: Life Sci Alliance. 2024 Jul 18;7(10):e202302516. doi: 10.26508/lsa.202302516 (PMC11258408; doi:10.26508/lsa.202302516)
Supplement: Supplementary file 5 [file LSA-2023-02516_TableS5.docx]

**Supplemental Table 5: List of antibodies**

| Name | Assay | Manufacturer | Catalog # | Concentration |
| --- | --- | --- | --- | --- |
| C/EBPβ | IF | Thermo | MA1-827 | 1:250 |
| pC/EBPβ | IF | Thermo | PA5-104823 | 1:60 |
| CSF1R | IF | R&D | AF3818 | 1:15 |
| CK8 | IF | DSHB | TROMA-1 | 1:100 |
| Cyclin D1  CK14 | IF  IF | Cell Signaling  BioLegend | 2978S  PRB-155P | 1:50  1:400 |
| PR | IF | Thermo | MA1-411 | 1:500 |
| BrdU | IF | Cell Signaling | 5292S | 1:500 |
| Ki67 | IF | abcam | Ab16667 | 1:100 |
| F4/80 | IHC | Biorad | MCCA497RT | 1:100 |
| C/EBPβ | WB | Santa Cruz | Sc-7962 | 1:1000 |
| β-actin | WB | Cell Signaling | 3700S | 1:1000 |
| CD24-FITC | FC | BD Biosciences | 563450 | 1:100 |
| CD29-APC | FC | Thermo Fisher | 48-0291-82 | 1:100 |

Abbreviations: IF, immunofluorescence; IHC, immunohistochemistry; FC, flow cytometry; WB, western blot
