# Supplementary material for: C/EBPβ deletion in macrophages impairs mammary gland alveolar budding during the estrous cycle
Source: Life Sci Alliance. 2024 Jul 18;7(10):e202302516. doi: 10.26508/lsa.202302516 (PMC11258408; doi:10.26508/lsa.202302516)
Supplement: Supplementary file 6 [file LSA-2023-02516_TableS6.docx]

**Supplemental Table 6: Primer sequences for qPCR**

| Gene symbol | Sense primer (5’-3’) | Antisense primer (5’-3’) |
| --- | --- | --- |
| *Cebpb* | TGATGCAATCCGGATCAA | CACGTGTGTTGCGTCAGT |
| *Ctnnb1* | GTTCGCCTTCATTATGGACTGCC | ATAGCACCCTGTTCCCGCAAAG |
| *Axin2* | ATGGAGTCCCTCCTTACCGCAT | GTTCCACAGGCGTCATCTCCTT |
| *Dkk1* | ATCTGTCTGGCTTGCCGAAAGC | GAGGAAAATGGCTGTGGTCAGAG |
| *Wnt5a* | GGAACGAATCCACGCTAAGGGT | AGCACGTCTTGAGGCTACAGGA |
| *Wnt4* | GAGAACTGGAGAAGTGTGGCTG | CTGTGAGAAGGCTACGCCATAG |
| *IL-6* | AGTCAATTCCAGAAACCGCTATGA | TAGGGAAGGCCGTGGTTGT |
| *Tnfa* | CTGTAGCCCACGTCGTAGC | TTGAGATCCATGCCGTTG |
| *Nos2* | GTCAACTGCAAGAGAACGGAGA | CTGAGAACAGCACAAGGGGTT |
| *Notch2* | ATGAACTGCCAGATGTCC | TGTCAGGCAAAATCAGAAAG |
| *Notch3* | AAGCGCGTCCTGGCATTGTCT | CCGCAGGGGCAGCAGTGGT |
| *Krt8* | AGTTCGCCTCCTTCATTGAC | GCTGCAACAGGCTCCACT |
| *Krt14* | ATCGAGGACCTGAAGAGCAA | TCGATCTGCAGGAGGACATT |
| 18s rRNA | GTAACCCGTTGAACCCCATT | CCATCCAATCGGTAGTAGCG |
